# The spike protein of the apathogenic Beaudette strain of avian coronavirus can elicit a protective immune response against a virulent M41 challenge

**DOI:** 10.1371/journal.pone.0297516

**Published:** 2024-01-24

**Authors:** Sarah Keep, Phoebe Stevenson-Leggett, Isobel Webb, Albert Fones, James Kirk, Paul Britton, Erica Bickerton

**Affiliations:** 1 The Pirbright Institute, Surrey, United Kingdom; 2 The Francis Crick Institute, London, United Kingdom; 3 School of Cellular and Molecular Medicine, Faculty of Life Sciences, The University of Bristol, Bristol, United Kingdom; Cairo University Faculty of Veterinary Medicine, EGYPT

## Abstract

The avian *Gammacoronavirus* infectious bronchitis virus (IBV) causes major economic losses in the poultry industry as the aetiological agent of infectious bronchitis, a highly contagious respiratory disease in chickens. IBV causes major economic losses to poultry industries across the globe and is a concern for global food security. IBV vaccines are currently produced by serial passage, typically 80 to 100 times in chicken embryonated eggs (CEE) to achieve attenuation by unknown molecular mechanisms. Vaccines produced in this manner present a risk of reversion as often few consensus level changes are acquired. The process of serial passage is cumbersome, time consuming, solely dependent on the supply of CEE and does not allow for rapid vaccine development in response to newly emerging IBV strains. Both alternative rational attenuation and cell culture-based propagation methods would therefore be highly beneficial. The majority of IBV strains are however unable to be propagated in cell culture proving a significant barrier to the development of cell-based vaccines. In this study we demonstrate the incorporation of a heterologous Spike (S) gene derived from the apathogenic Beaudette strain of IBV into a pathogenic M41 genomic backbone generated a recombinant IBV denoted M41K-Beau(S) that exhibits Beaudette’s unique ability to replicate in Vero cells, a cell line licenced for vaccine production. The rIBV M41K-Beau(S) additionally exhibited an attenuated *in vivo* phenotype which was not the consequence of the presence of a large heterologous gene demonstrating that the Beaudette S not only offers a method for virus propagation in cell culture but also a mechanism for rational attenuation. Although historical research suggested that Beaudette, and by extension the Beaudette S protein was poorly immunogenic, vaccination of chickens with M41K-Beau(S) induced a complete cross protective immune response in terms of clinical disease and tracheal ciliary activity against challenge with a virulent IBV, M41-CK, belonging to the same serogroup as Beaudette. This implies that the amino acid sequence differences between the Beaudette and M41 S proteins have not distorted important protective epitopes. The Beaudette S protein therefore offers a significant avenue for vaccine development, with the advantage of a propagation platform less reliant on CEE.

## Introduction

The highly contagious *Gammacoronavirus* infectious bronchitis virus (IBV) causes infectious bronchitis in domestic fowl, resulting in significant economic losses to poultry industries worldwide [1]. IBV has a positive sense RNA genome encoding four structural proteins known as the spike (S), membrane (M), envelope (E) and nucleocapsid (N) proteins [2]. Infection primarily manifests with respiratory symptoms including snicking and tracheal rales, however some strains also cause pathology in the kidneys and oviducts and recent literature suggests replication in gastrointestinal and lymphoid tissue [3–5]. Infection with IBV induces extensive damage to the ciliated cells that line the trachea, causing cessation of ciliary movement, otherwise referred to as ciliostasis. This can lead to secondary bacterial infections responsible for the mortality associated with the disease in poultry practice [1, 6–8]. Reductions in weight gain and egg production in infected broilers and laying birds, respectively, are also observed. Decreases in egg production are the result of reductions in laying rates, deformed shells and eggs laid without shells [1, 9]. Vaccine-based IBV infection control is therefore a major concern for animal welfare, economics and in protecting global food security.

Vaccines against IBV are available, mainly as live-attenuated viruses, which are produced by serial passage of a field strain, typically between 80 and 100 times [10, 11]. A fine balance must be achieved between attenuation and retention of immunogenicity. The molecular mechanisms of both characteristics are not well understood. The exact vaccination regimes used depends on the type of bird being vaccinated. Broilers are typically vaccinated with live-attenuated vaccines and laying birds generally receive live-attenuated IBV vaccines followed by inactivated vaccines prior to the laying period [9]. Regardless of the regime, vaccine induced protection is often short-lived and cross protection between the many different strains of IBV, which are classified by both genotype and serotype, is poor. Chickens are therefore vaccinated with several separate IBV vaccines to maximise the chances of generating immunity against relevant viruses, using strains from different serotypes and/or genotypes, depending on geographic distribution of strains [9, 12, 13]. Investigation into sequential vaccination has shown that using vaccines from different serotypes does increase the chance of achieving cross-protection against different IBV types [14, 15]. Classifying IBV strains into protectotypes, a classification that groups IBV strains together on the basis of whether infection results in a protective immune response against another defined IBV and is independent of both serotype and genotype, aims to increase efficacy of vaccine regimes however the process is labour intensive, time consuming and expensive [16].

Vaccine induced protection against IBV is mainly associated with the S protein, a large heavily glycosylated protein that mediates viral entry [12, 13, 17]. This association is not only for IBV but also other coronaviruses, including severe acute respiratory syndrome coronavirus (SARS-CoV), Middle East respiratory syndrome coronavirus (MERS-CoV) and SARS-CoV-2 in which the S protein has formed the basis of vectored vaccines and therapeutics [18–20] The S protein is divided into two subunits, S1, the globular head which contains two putative receptor binding domains, denoted S1-NTD and S1-CTD and the S2, the stalk that mediates virus to cell and cell to cell fusion [21]. The majority of neutralising IBV antibodies are associated with the S1 subunit [22–25]. Cross protection between the genetically or antigenically related IBV strains is difficult to predict highlighting the complicated relationship between genotype, serotype and protectotype. The consensus assumption is however one of a negative correlation; the greater the difference in amino acid identity of the S protein between the vaccine and challenge strain, and specifically differences in the S1, results in an increasing likelihood of poor levels of cross protection [26, 27]. Cross protection is however not that clear cut with several studies demonstrating a cross protective immune response between genetically and/or antigenically divergent, unrelated strains [15, 27–31]. There are also examples of related IBV strains belonging to the same serotype that cannot induce a protective immune response [32]. Cross protection and consequently prediction of vaccine efficacy is not a problem unique to IBV. The constant evolution of SARS-CoV 2 continues to present concerns regarding whether currently licenced vaccines based on the original Wuhan S gene sequence [33–35] can protect against emerging variants [36]; a particular concern as current vaccines have limitation with regards to blocking viral replication and transmission [37, 38].

The widely researched attenuated Beaudette strain of IBV has long been considered to be poorly immunogenic [39, 40] and it has never been used as a vaccine. It is the first strain of IBV, and the first coronavirus, to be isolated in 1937 and has since been extensively passaged in both cell culture and *in ovo*, reportedly upwards of 200 times although the exact passaging history is unknown [32, 41]. Although both Beaudette and the pathogenic M41 strain belong to the Massachusetts serotype and GI-I genotype [42], Beaudette does not induce protection against a pathogenic M41 challenge [32]. One hypothesis is that extensive passaging, or otherwise termed over passaging, has negatively affected immunogenicity; such a scenario has been associated with reduced vaccine efficacy [11]. The other hypothesis centres on differences between the S proteins. There are 26 amino acid residue differences between the S1 sequence of the Beau-CK (accession number AJ311317) and M41-CK (accession number MK7288751) and it has been hypothesised that these differences have removed or distorted cross protective epitopes [12]. It should be noted that the majority of these differences are located in the three hypervariable regions, HVR1, HVR2, HVR3, of the S1 subunit, that have been hypothesized to be associated with cross protective epitopes [42–45].

Previously we generated a recombinant IBV (rIBV) denoted BeauR-M41(S) in which the ectodomain sequence of the S gene, within the Beaudette molecular clone Beau-R, was replaced with that of the corresponding sequence derived from M41-CK [46]. The rIBV remained attenuated and birds vaccinated with rIBV BeauR-M41(S) were protected from M41-CK challenge, however the protection induced fell short of the standards set out by the European Pharmacopoeia [32, 47]. This may have been the consequence of the inability of Beau-R to replicate efficiently *in vivo* which may be associated with its temperature sensitive replication phenotype [48]. Equally it may also be the consequence of other antigenic factors located elsewhere in the genome. The N protein has for example been shown to have both T and B cell epitopes [49, 50]. This therefore raised the question of, if presented in a different genomic background, whether the Beaudette S protein could elicit a protective immune response.

The S protein of Beaudette, and that of rIBV Beau-R has an advantageous characteristic in that it confers Beaudette’s unique ability to replicate in Vero cells; a cell line licensed for vaccine manufacturing [51, 52]. The Beaudette S protein, if immunogenic, could therefore offer an avenue for IBV vaccine manufacturing in cell culture, as done with other viral pathogens including influenza, rabies and polio [52, 53]. Vaccine production in Vero cells not only offers a platform less reliant on CEE but alongside rational attenuation also allows for a more rapid response to emerging strains, the importance of which has been emphasised not only by emergence of novel IBV strains [54, 55] but also during the SARS-CoV-2 pandemic. Most field strains of IBV do not replicate in cell culture, which has so far proved to be a limitation in development of cell-based IBV vaccines. To assess the potential of a cell adapted vaccine, we generated a rIBV based on a pathogenic molecular clone of the M41-CK strain of IBV, denoted M41-K. Using a vaccinia virus based reverse genetics system the ectodomain sequence of the S gene of M41-K was replaced with the equivalent sequence from Beau-R generating the rIBV M41K-Beau(S). *In vitro* assays demonstrated the Beau-R S had conferred the ability to replicate in Vero cells and had also conferred an attenuated *in vivo* phenotype. Vaccination with either M41K-Beau(S) or M41R-Beau(S), a virus containing additional attenuating mutations in non-structural proteins (nsp) 10 and 14 [56] induced complete protection against M41-CK challenge, demonstrating that the Beaudette S protein is immunogenic. Immunogenicity was irrespective of whether the Beaudette S protein was presented from a virulent or attenuated M41 genetic background. Our results demonstrate that the amino acid differences between the M41 and Beaudette S proteins are not the cause of the inability of Beaudette to act as a vaccine, opening up the highly significant possibility of the using the Beaudette S protein for future vaccine development.

## Materials and methods

### Ethics statement

Primary cells and tracheal organ cultures as well as all *in vivo* experiments were performed in strict accordance with UK Home Office regulations concerning the use of animals in scientific procedures by trained personnel. All experiments and the use of animals for the preparation of primary cells and tracheal organ cultures were approved by the Animal Welfare Ethical Review Body at The Pirbright Institute.

### Cells and viruses

Primary chick kidney (CK) cells were prepared by the Central Services Unit (CSU) at The Pirbright Institute (Surrey, UK) using kidneys from specific pathogen free (SPF) 2–3-week-old Rhode Island Red (RIR) chickens following a method previously described [57]. Cells were seeded in tissue culture plates at 0.8 x 10^6^ cells per ml three days prior to infection with IBV. Vero cells, a mammalian continuous cell line derived from the kidney of a vervet monkey [58] were also provided by CSU at The Pirbright Institute. The cells were originally purchased from ECACC (www.culturecollections.org.uk Cat no. 84113001) in 1997. Vero cells were seeded at 0.8 x 10^5^ cells/ml to achieve confluency three days post seeding. Of note for all cell types, 6 well tissue culture plates were seeded with 3 ml per well and 12 well tissue culture plates 2 ml per well. All cells were incubated at 37°C, 5% CO_2_.

Tracheal organ cultures (TOCs) were prepared from SPF 19-day old RIR chicken embryos [59]. Embryos were culled by decapitation and trachea were sectioned using a microtome. Individual 1 mm sections (rings) were placed in glass test tubes in 1 ml of TOC growth medium [59] and incubated overnight at 37°C in a rotating incubator (no CO_2_). After 24 hours (h), each TOC was assessed for ciliary activity and those exhibiting less than 90% ciliary activity were discarded and those exhibiting more than 90% activity were retained and used for infection the following day. SPF chicken embryonated hens’ eggs (CEE) were obtained from Valo BioMedia GmbH (Germany), set at The Pirbright Institute and used to propagate virus stocks at 10 days old.

M41-CK (GenBank accession number MK728875.1) is a pathogenic IBV isolate belonging to the Massachusetts serotype and has been passaged multiple times in cell culture [60]. M41-K is a molecular clone of M41-CK, generated by reverse genetics [56]. Both M41-CK and M41-K can be propagated in primary CK cells. Beau-R is a recombinant molecular clone of Beaudette-CK (GenBank accession number AJ311317), also belonging to the Massachusetts serotype [61]. Beaudette viruses exhibit an extended tropism *in vitro* and are able to infect and replicate successfully in Vero cells as well as CK cells. The 4/91 strain (GenBank accession number JN192154) used in this study was a gift from Intervet and is a pathogenic field strain unable to replicate in cell culture. This strain can only be propagated in TOCs or CEE. All virus stocks were propagated in CEE and viral titres were determined by titration in CK cells or TOCs to determine the plaque forming units per ml (PFU/ml) or ciliostatic dose 50 (CD_50_/ml). All nucleotide positions relate to M41-CK.

### Reverse genetics and recombinant virus rescue

Recombinant viruses were generated according to previously described reverse genetics system protocols [62, 63]. In all rIBVs the signal sequence (SS), transmembrane (TM) domain and cytoplasmic tail (CT) of the S gene is derived from M41-K sequence, nucleotides 20356–20406 and 23677–23817 respectively. The former is to preserve the sequence of the end of nsp 16 as it overlaps with the S open reading frame. The latter two preserve interactions with the other structural proteins. To generate M41K-Beau(S) and M41K-4/91(S), the ectodomain sequence of the M41-K S gene, nucleotides 20405–23676, were replaced with the corresponding sequence from either Beau-R or 4/91. The rIBV M41R-Beau(S) contains additional mutations: Pro85Leu in nsp 10, Val393Leu in nsp 14, Leu183Ile in nsp 15 and Val209Ile in nsp 16 which are associated with in an attenuated phenotype *in vivo* (56). All rIBVs were recovered in CK cells as described previously [63]. The rIBVs M41K-Beau(S) and M41R-Beau(S) were passaged in CK cells twice before a stock was generated in CEE. The rIBV M41K-4/91(S) was passaged in CEE and a stock generated at passage two. Virus presence and gene identities were confirmed by RT-PCR and Sanger sequencing using primers targeting the S gene and flanking sequences (5ʹ-AATAATGGCAATGATGAC-3ʹ, 3ʹ-AACTGCCACAAACATACTGC-5ʹ, 5ʹ-CATCAAAATCACTAATGG-3ʹ, 3ʹ-AGGGATCAAATACTTCTGTG-5ʹ).

### Assessment of replication kinetics in cell culture

CK and Vero cells seeded in 6 well plates were washed once in phosphate buffered saline (PBS) and infected with 10^4^ or 0.5 x 10^3^ PFU (MOI ~0.001) of IBV in a 500 μl volume of BES [N,N-bis(2-hydroxyethyl)-2-aminoethanesulphonic acid; Sigma] medium [63]. Infected cells were incubated at 37°C (5% CO_2_) for 1 h before two washes in PBS to remove residual unbound virus particles. Per well, 3 ml of BES medium was added and cells were incubated at 37°C (5% CO_2_). Supernatant was harvested at 1, 24, 48 and 72 hours post-infection (hpi) and the quantity of infectious progeny determined by plaque assay in triplicate in CK cells.

### Assessment of replication kinetics in *ex vivo* TOCs

In replicates of either three or four, TOCs (three individual rings) were placed in 100 mm x 16 mm borosilicate glass medium wall rimless test tubes (Fisherbrand) and were washed once in PBS prior to inoculation with 10^4^ CD_50_ of IBV or rIBV. TOCs were incubated at 37°C (no CO_2_) in an upright test tube rack for 1 h. TOCs were washed once in PBS and 1 ml of TOC infection medium (0.5 x EMEM, 75 μM α-methyl-D-glycoside, 40 μM HEPES, 0.1 Sodium Bicarbonate, 10 U/ml penicillin, 10 μg/ml streptomycin, all obtained from Sigma) was added to each tube. TOCs were incubated at 37°C (no CO_2_) in a rotating incubator, 8 revolutions per hour. Supernatant was harvested at 1, 24, 48, 72 and 96 hpi. The quantity of infectious progeny was determined either via plaque in CK cells or via titration in *ex vivo* TOCs as previously described [59].

### Assessment of ciliary activity in *ex vivo* TOCs

In replicates of ten, TOCs were washed once in PBS and infected with 10^4^ PFU or equivalent CD_50_ of IBV or rIBV in a volume of 0.5 ml, diluted in TOC infection medium. TOCs were incubated at 37°C (no CO_2_) in a rotating incubator, 8 revolutions per hour. Ciliary activity was assessed under a light microscope prior to infection and then at 24, 48, 72 and 96 hpi. TOCs were assigned a score between 0 and 4, with 1 representing 25% ciliary activity and 4 representing 100%. The mean average score of the 10 replicates was calculated.

### *In vivo* experiments

All *in vivo* experiments were performed in strict accordance with Home Office regulations concerning the use of animals in scientific procedures by trained personnel. Training was provided to all personnel on animal care, welfare and handling. SPF RIR chicks used in Experiment 1 and 4 were hatched and reared in the Poultry Production Unit (PPU) at The Pirbright Institute, Compton Laboratory and housed during the experiment in the Experimental Animal House (EAH) on the same site. SPF RIR chicks in Experiments 2 and 3 were purchased from the National Avian Research Facility (NARF, University of Edinburgh), delivered to The Pirbright Institute (X24684464) at 1 day of age, reared and housed during the experiment in the Biological Services Unit (BSU). Chickens in all experiments were housed in raised floor pens with enrichment including perches and were monitored at least twice daily throughout the duration of the experiment. Humane endpoints for all experiments were as follows: 1) sitting alone and not evading capture: the bird will be euthanised immediately, 2) respiratory distress e.g. excessive gasping: the bird will be euthanised immediately, 3) snicking and/or rales for seven days in total: the bird will be euthanised on the beginning to the 7th consecutive day and 4) excess drinking for more than two days as indicated by a fluid filled crop: the bird will be euthanised at the beginning of the 3rd consecutive day. None of the birds in any of the studies reached the humane endpoints and all except 1 were culled as part of scheduled experimental culls. One bird during experiment 3 was found dead; post-mortem analysis showed that the death was not related to procedures used in the experiment or IBV. Total duration of each experiment is as follows: Experiment 1 and 4, one week, Experiment 2, two weeks and Experiment 3, six weeks.

#### Experiments 1, 2 and 4: Assessment of pathogenicity

Chickens were randomly assigned to groups and housed in groups of 12 (Experiment 1 and 4) or 15 (Experiment 2). At 8 days of age, birds were inoculated with 100 μl of PBS containing (Experiment 1) 10^4^ PFU of either M41K-Beau(S), M41-K or M41-CK, (Experiment 2) 10^5^ PFU or equivalent CD_50_ of either M41K-4/91(S), M41-K or 4/91, or (Experiment 4) with 10^5^ PFU of M41R-Beau(S), M41-R or M41-CK by the intraocular (eyedrop) and intranasal route. Approximately 25 μl of inoculum was administered per eye and per nasal cavity. In both experiments a mock group, containing the equivalent animal numbers as the test groups, was inoculated with 100 μl PBS by the same method. Total animal numbers for each experiment were therefore as follows: experiment 1, 48 chicks, experiment 2, 60 chickens and experiment 4, 60 chickens. Chickens were assessed for IBV-induced clinical signs including snicking and rales from either day 2 or day 3 to day 7 post-infection (pi) and in experiment 2 on day 14 pi. Three (Experiment 1 and 4) or five (Experiment 2) randomly selected birds from each group were culled on days 4 and 6 pi by cervical dislocation by a trained member of The Pirbright Institute Animal Services. The trachea, eyelid, kidney, beak and lung were harvested and sections stored in 500 μl RNA Later solution (Thermo Fisher) and in 500 μl PBS. All remaining birds were culled on day 7 pi in Experiment 1 and 4 and day 14 pi in Experiment 2. During the latter, blood was collected and processed for serum. At days 4 and 6 pi, ciliary activity was assessed in the extracted trachea by a person blinded to the animal number and group, as previously described [26, 27]. The data regarding the control groups, mock, M41-CK and M41-R for Experiment 4 have been published previously [56].

#### Experiment 3: Vaccine-challenge experiment

A total of 120 chickens were randomly assigned to groups and housed in four groups of 30. At 8 days of age, birds were inoculated with 100 μl of PBS containing 10^4^ PFU of either M41K-Beau(S) or M41R-Beau(S) by the intraocular (eyedrop) and intranasal route. Two mock groups, also containing 30 birds each, were inoculated with 100 μl PBS by the same method. Chickens were assessed for IBV induced clinical signs, including rales and snicking from day 3 to day 7 post-vaccination (pv). On day 14 pv all birds were bled via wing prick, and the serum isolated. Due to the limited quantities of serum obtained, serum from all birds in each group were pooled together. On day 27 pv, all birds were challenged via the intraocular and intranasal route with either 100 μl of PBS containing 10^4^ PFU of M41-CK or for mock challenge, 100 μl of PBS. Of note, one mock vaccinated group was challenged with M41-CK, and the other mock vaccinated group was challenged with PBS. Birds were assessed for IBV-induced clinical signs from day 3 to day 7 post-challenge (pc). On days 2 and 4 pv and days 4 and 6 pc, six randomly selected birds were culled by cervical dislocation by a trained member of The Pirbright Institute Animal Services. From each bird, samples of tissues including eyelid and trachea were collected and stored in both 500 μl RNA Later solution (Thermo Fisher) and in 500 μl PBS to allow for potential assessment via multiple assays including RT-PCR analysis and virus isolation respectively. On day 4 pv and on both days 4 and 6 pc tracheal ciliary activity of each extracted trachea was assessed by a person blinded to the animal number and group, following the method previously described [26, 27]. All remaining birds were culled at day 14 pc. At every time point, after each bird was culled, the bird was decapitated, and blood collected via the exposed blood vessels in the neck. The collected blood was allowed to clot to allow for serum collection. The data regarding the control groups, mock vaccinated/M41-CK challenged and mock vaccinated/mock challenged have been published previously [64].

#### Virus re-isolation in chicken embryonated eggs (CEE)

Samples of trachea, eyelid, beak, lung, and kidney harvested during pathogenicity Experiments 1 and 2, and trachea harvested as part of Experiment 3 stored in PBS were homogenised in the PBS for 2–4 minutes using a TissueLyser II and a 5 mm stainless-steel bead (QIAGEN). Homogenised samples were clarified by centrifugation to remove tissue debris and 100 μl supernatant was used to infect 10-day old embryonated SPF CEEs using a 1 ml syringe and 25G needle. Eggs were incubated in a tilting incubator at 37°C for 24 h, after which they were transferred to 4°C for a minimum of 4 h. Allantoic fluid was harvested from each egg, clarified by centrifugation and 170 μl used for RNA extraction following the RNA Clean-Up protocol in the RNeasy Mini Kit (QIAGEN). The presence of IBV derived RNA indicative of the presence of infectious progeny was determined by RT-PCR analysis using primers specific for the 3′UTR [65].

#### Assessment of viral load in *ex vivo* TOCs

Samples of upper eyelid and trachea were homogenised in the 500 μl volume of PBS they were stored in and serially diluted in TOC infection media (1 X EMEM containing 7.5% methyl α D-glucopyranoside, 1% L-Glutamine, 5% Hepes, 0.5% Nystatin and 0.1% Penicillin/Streptomycin, all obtained from Sigma). The quantity of infectious virus was determined via titration in *ex vivo* TOCs as previously described [59]. Viral titres were calculated using the Reed-Muench method for end point titre calculations [66] and presented as CD_50_/ml.

#### IBV-specific antibody detection by ELISA

Samples of serum collected from birds in Experiment 3 (vaccination/challenge) were heat-inactivated at 56°C for 30 minutes (min) and diluted 1:80 in Sample Diluent (BioChek). Levels of IBV-specific antibody were measured in each sample using a commercial IBV ELISA kit (BioChek) following the manufacturer’s instructions. Sample:positive (S/P) ratios were calculated using the following equation: [(mean sample–mean kit negative)/(mean kit positive–mean kit negative)]. S/P ratio values greater than 0.2 were considered positive for IBV-specific antibodies. Positive and negative controls were included in each independent test plate. Note, the serum samples from day 14 pv were pooled for this analysis due to the small quantities collected.

#### Quantification of neutralising antibody

Levels of neutralising antibody against M41-CK in the serum of each bird from Experiment 3 were analysed by plaque reduction assay in CK cells. Heat-inactivated serum samples were diluted two-fold in BES medium in triplicate, from 1:5 to 1:160. An equal volume of BES medium containing 10^3^ PFU of M41-CK was added to each dilution of serum. Virus-serum mixtures were incubated for 30 min at room temperature on an orbital shaker. Each mixture was serially diluted in a 10-fold dilution series in BES medium and titrated on CK cells to determine the viral titre after incubation with serum. Neutralising antibody titres were calculated from three biological repeats using the Reed-Muench method [66]. Average neutralising antibody titres were displayed as plaque reduction neutralisation titre 50 values (PRNT_50_).

### Sanger sequencing

PCR products and primers were prepared for Sanger sequencing in accordance with Source Bioscience guidelines (Cambridge, UK). Sequencing reads were analysed using Staden software and alignments were built using BioEdit.

### Statistical analysis

All statistical analyses were performed using GraphPad Prism version 8.0. Normality and the standard deviation of each dataset were assessed prior to each statistical test. A Two-Way ANOVA statistical analyses was performed on all data sets generated from all growth kinetic assays, ciliary activity assays in *ex vivo* TOCs as well as ciliary activity in tracheas extracted from *in vivo* experiments. A One-way ANOVA statistical analyses was performed on data sets regarding viral load in tissues harvested from *in vivo* experiments and neutralising antibody. Unless otherwise specified Tukey’s test was used for analyses of multiple comparisons.

## Results

### The S protein of Beaudette confers the ability to replicate in Vero cells

Until the ability to generate molecular clones of IBV, using reverse genetics, it had not been possible to investigate the actual role of the Beaudette S protein in pathogenicity and determine conclusively whether the Beaudette S protein could result in an attenuated phenotype or whether it could result in a protective immune response. Using a vaccinia virus based reverse genetics system [63] the ectodomain sequence, between nucleotides 20405 to 23676 of the S gene from the pathogenic rIBV M41-K [56], a pathogenic clone of the M41-CK strain, was replaced with the equivalent sequence from attenuated rIBV Beau-R [61]. Beau-R is a molecular clone of Beau-CK [61]; the sequence of the S gene is identical between Beau-R and Beau-CK. In line with our previous research [46, 67] the signal sequence (SS), transmembrane domain (TM) and cytoplasmic tail (CT) of the M41-K S were not replaced in order to both retain the M41 derived sequence at the end of nsp 16 and to conserve the interactions between S protein and the other viral proteins [68, 69]. Two isolates of rIBV M41K-Beau(S) were recovered in primary CK cells and passaged three times in CK cells before passaging twice in CEE, with stock virus generated at egg passage 2. The presence of a successfully recovered rIBV was confirmed by observation of cytopathic effect and through RT-PCR targeting both the 3’UTR and S gene. The sequence of the S gene and adjacent sequences were confirmed by Sanger sequencing; the sequences of both isolates of M41K-Beau(S) were as expected. One isolate was therefore selected and taken forward for future experiments. As it was possible that the replacement of the S ectodomain gene sequence with that of heterologous IBV S sequence could alter the *in vitro* and *in vivo* phenotypes, including pathogenicity, of the resulting rIBV rather than the nucleotide sequence of the gene itself, a second rIBV was generated. Following a similar methodology, the ectodomain of the S sequence of rIBV M41-K was replaced with the equivalent sequence derived from the pathogenic IBV strain 4/91, resulting in the rIBV M41K-4/91(S). IBV 4/91 belongs to a different serotype and genotype to Beaudette and M41, with only 81.7% amino acid identity to the M41 S protein sequence; whereas the Beaudette and M41 S proteins have 95.4% amino acid identity and belong to the same, Massachusetts, serotype. One isolate of the rIBV M41K-4/91(S) was successfully rescued in CK cells, serially passaged in CEE to increase the amount of virus present (as IBV 4/91 does not productively replicate in CK cells), and a stock was generated at egg passage 2. The S gene of the rIBV was sequenced; no additional mutations were identified.

**Fig 1:**
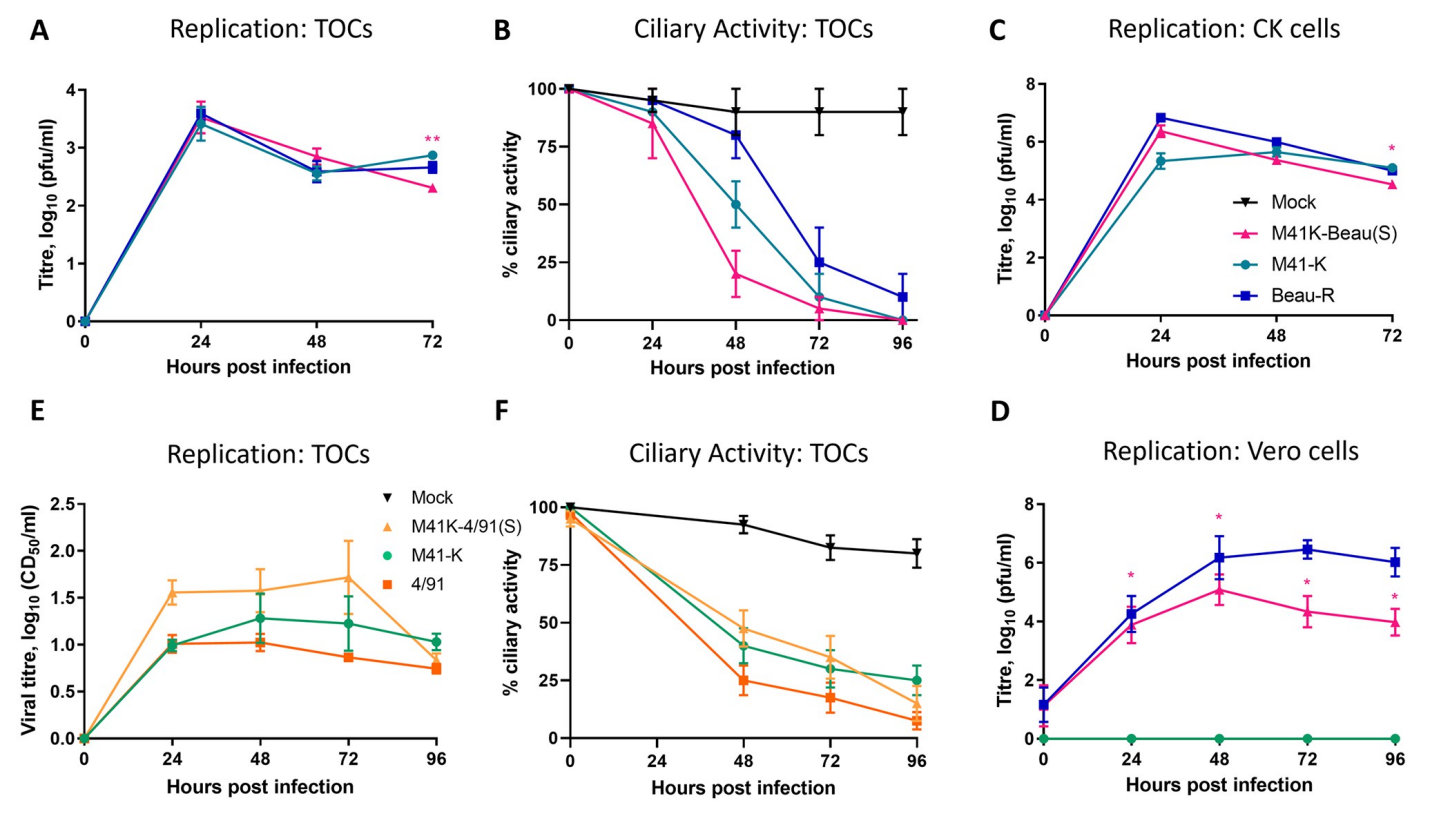
The incorporation of a heterologous S gene has resulted in altered tropism *in vitro*. (A, B, E, F) *Ex vivo* TOCs, (C) CK cells and (D) Vero cells were inoculated with 10^4^ PFU or CD_50_ equivalent dose of either (A-D) M41-K, Beau-R or M41K-Beau(S) or (E-F) of M41-K, 4/91 or M41K-4/91(S). (A, C, D, E) Supernatant was collected at 24-hour intervals and titrated on (A, C, D) CK cells or (E) in *ex vivo* TOCs. (B, F) The percentage ciliary activity was assessed at 24-hour intervals. Note: TOCs were inoculated in replicates of 10, and mock infected TOCs were inoculated with media only. (A–F) Each data point represents the average titre from three independent experiments with error bars representing SEM. Statistical differences, * p<0.05 and ** p<0.05 were assessed by Two-Way ANOVA followed by Tukey’s test for multiple comparisons. (B, F) Statistical differences identified were between all viruses and mock from 24 to 96 hpi; no differences were identified between the viruses. (A, C, D) Only statistical differences between M41K-Beau(S) and M41-K are displayed. Additional statistical differences are as follows: (C) M41-K vs Beau-R, 24 hpi and (D) M41-K vs Beau-R, 24–96 hpi.

To confirm whether the heterologous S containing rIBVs retained their ability to replicate and to determine if the Beaudette S had conferred the ability to replicate in Vero cells, the replication kinetics of both rIBVs were assessed *in vitro* and in *ex vivo* TOCs (Fig 1). In both CK cells and *ex vivo* TOCs, rIBV M41K-Beau(S) displayed kinetics similar to both the S gene donor strain, Beau-R and the genomic backbone M41-K (Fig 1A–1C). Although it appeared that rIBV M41K-Beau(S) resulted in the quickest decline in ciliary activity, statistical significance was not reached (Fig 1B). M41K-Beau(S), unlike M41-K demonstrated the ability to replicate in Vero cells, however replication was reduced in comparison to Beau-R (Fig 1D). Whilst M41-K can replicate productively in CK cells, 4/91 exhibits a more restricted tropism, only able to replicate in CEE and TOCs. As expected, with the addition of the 4/91 derived S protein, rIBV M41K-4/91(S) replication was undetectable in CK cells, by both RT-PCR and plaque assay therefore, replication kinetics were assessed only in *ex vivo* TOCs (Fig 1E and 1F). The recombinant virus replicated in TOCs to slightly higher titres than both M41-K and 4/91 although the differences were not statistically significant. Reductions in tracheal ciliary activities following infection were also investigated and were found to be comparable to both M41-K and 4/91.

### The Beau-R S confers an attenuated *in vivo* phenotype

**Fig 2:**
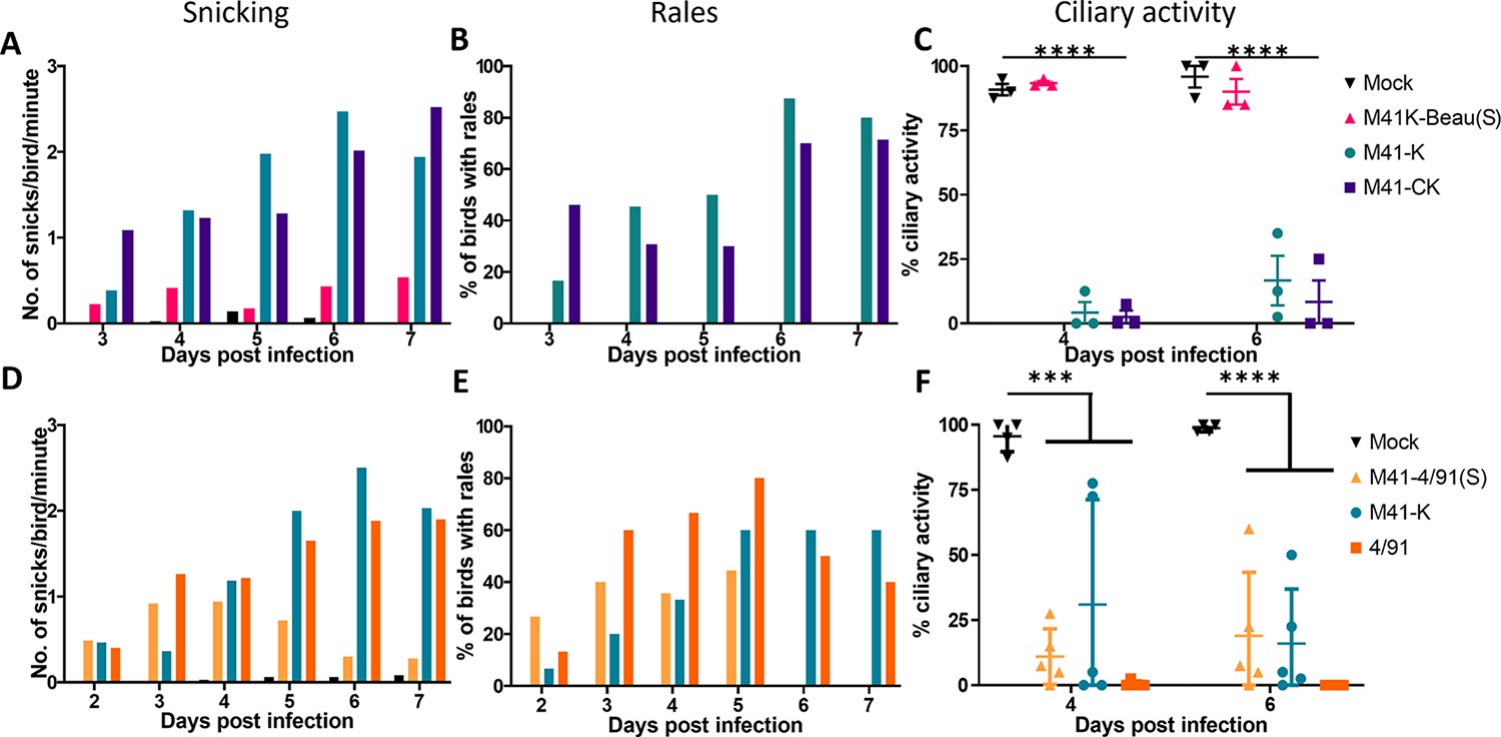
M41K-Beau(S) is attenuated *in vivo*. At 8 days of age, four groups of (A-C) 12 or (D-F) 15 SPF RIR chicks were inoculated via the intraocular and intranasal route with 10^5^ PFU or equivalent CD_50_ dose of either (A-C) M41K-Beau(S), M41-K or M41-CK or (D-F) with M41K-4/91(S), M41-K or 4/91. Mock infected birds were inoculated with PBS. Clinical signs were observed from either day 2 or 3 to day 7 post-infection. (A, D) The average number of snicks per bird per minute was calculated for each group based on counts from two persons. (B, E) The percentage of birds exhibiting rales in each group was calculated. (C, F) Ciliary activity in the trachea was measured on days 4 and 6 post-infection. Trachea were extracted from three or five randomly selected birds on each day and sectioned into 10 x 1 mm rings. Each ring was analysed under a light microscope and assigned a score between 1 and 4 depending on the proportion of cilia beating. 1 = 25% beating, 2 = 50%, 3 = 75%, 4 = 100%. Each data point represents the average percentage ciliary activity of each bird. Error bars represent SD. Statistical differences were assessed using a Two-Way ANOVA followed by Tukey’s test for multiple comparisons and are highlighted by ***(p<0.0005) and **** (p<0.0001).

The pathogenic phenotypes of rIBVs M41K-Beau(S) and M41K-4/91(S) were assessed in two separate *in vivo* experiments (Fig 2). In the first, groups of 12 RIR SPF chickens were inoculated with 10^5^ PFU of either M41-K or M41K-Beau(S) via the intraocular/intranasal route or mock inoculated with PBS. Beau-R was not included in the experimental design as its *in vivo* phenotype is well characterised [32, 48, 56]. In the second, groups of 15 RIR chickens were infected with 10^5^ PFU or 5.5 CD_50_ equivalent of either M41-K, 4/91 or M41K-4/91(S), or mock infected with PBS by the intraocular and intranasal route. Clinical signs were observed, including snicking (Fig 2A and 2D) and rales (Fig 2B and 2E), from day 2/3 to day 7 pi. As expected, in both experiments very low levels of snicking and no rales were observed in the mock-infected birds. The pathogenic control group in experiment 1 M41-K, exhibited snicking and rales on all days pi peaking on day 6 (Fig 2A and 2B). In experiment 2 snicking in the M41-K and 4/91 pathogenic control groups peaked at day 6 and 7 respectively (Fig 2D and 2E); rales for both groups peaked at day 5 pi. Chickens infected with M41K-Beau(S) exhibited clinical signs comparable to the mock group, with very few snicks detected and no rales suggesting that infection with M41K-Beau(S) does not result in clinical disease symptoms. Chickens infected with M41K-4/91(S) however exhibited snicking and rales; the latter peaking on 5 pi. The pattern of snicking and rales appeared to peak earlier than both M41 and 4/91 with no rales observed on days 6 or 7 pi.

*In vivo*, IBV primarily replicates in tracheal epithelial cells causing a reduction in tracheal ciliary activity; the loss of tracheal ciliary activity is the established marker of IBV pathogenicity [14, 48, 56]. Ciliary activity in the tracheas harvested from three (Experiment 1) or five (Experiment 2) randomly selected birds in each group was assessed on days 4 and 6 pi (Fig 2C and 2F). As expected, the average ciliary activities in the M41-K infected birds were reduced to less than 50% on days 4 and 6 pi in Experiment 1. In the tracheas extracted from birds inoculated with M41K-Beau(S), the ciliary activities were comparable to those observed in the mock infected group, greater than 75% on both days 4 and 6 pi (Fig 2C). The retention of ciliary activity alongside the lack of IBV induced clinical signs demonstrated that M41K-Beau(S) exhibited an attenuated phenotype *in vivo*.

In Experiment 2 on day 4 pi, ciliary activity in all the 4/91 infected birds analysed was completely abolished (Fig 2F). In the M41-K infected birds, ciliary activity was reduced to an average of 30% but there was variation between birds, with one retaining 75% activity. Ciliary activity in the M41K-4/91(S) birds was substantially reduced, with little variation observed between birds, more akin to that observed in the 4/91 group. On day 6 pi, a greater variation in ciliary activities between birds infected with M41K-4/91(S) were observed, with one bird retaining up to 60% activity. This was more comparable to that observed in trachea extracted from M41-K infected birds. In contrast, at day 6 pi ciliary activity in the 4/91 infected birds remained at zero. The observed clinical signs and reduction in tracheal ciliary activity demonstrate that M41K-4/91(S) exhibited a pathogenic phenotype *in vivo*. This indicates that the observed attenuated *in vivo* phenotype exhibited by M41K-Beau(S) (Fig 2) is not simply the result of a large heterologous nucleotide exchange but is the consequence of the incorporation of the Beau-R S gene.

### Beaudette S permits replication in tracheal tissue *in vivo*

**Fig 3:**
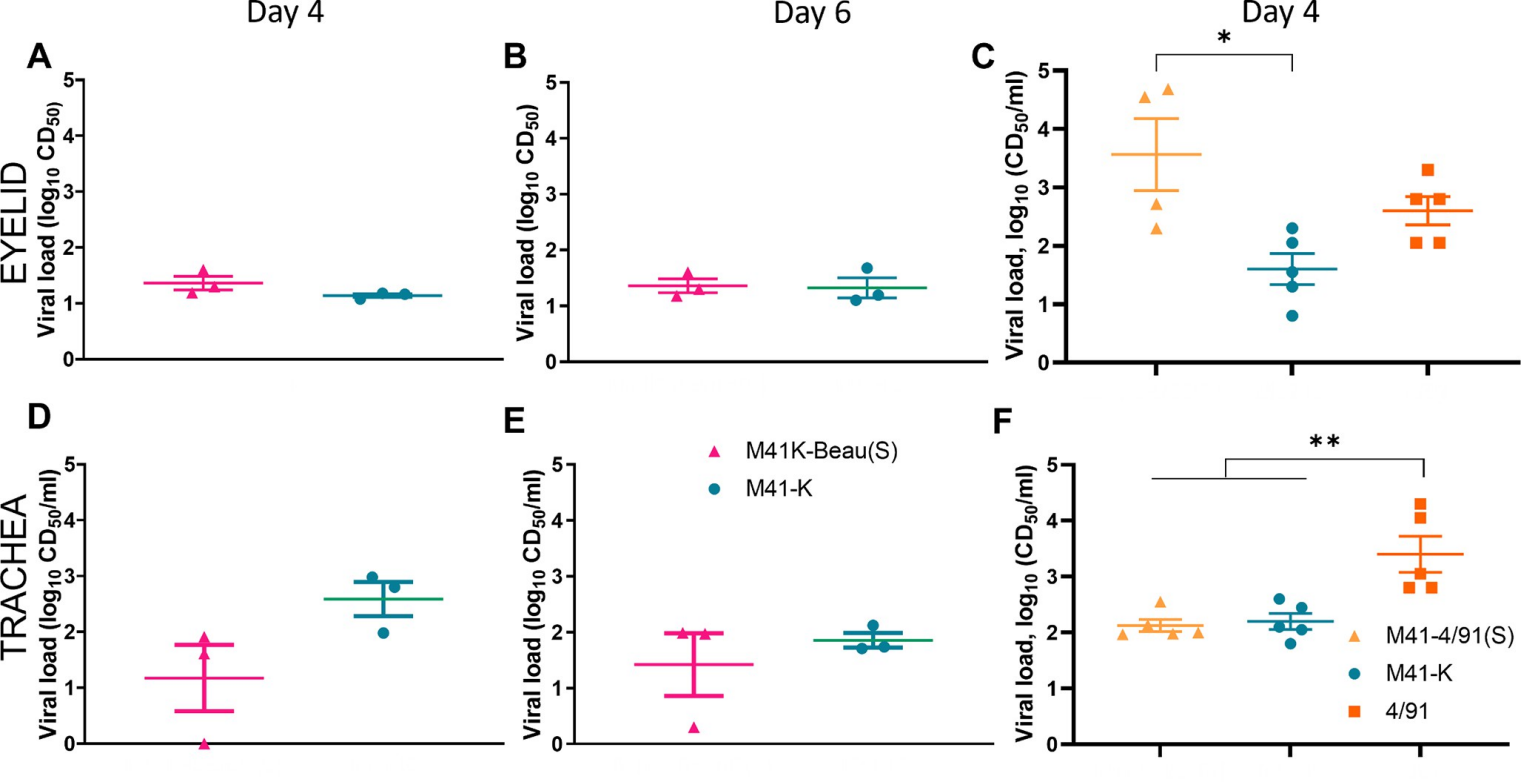
Viral load in trachea and eyelid tissue is comparable between M41K-Beau(S) and M41-K. Samples of homogenised (A–C) eyelid and (D–F) trachea harvested on days 4 and 6 post-infection for experiment 1 and day 4 only for experiment 2 were titrated in *ex vivo* TOCs. Unfortunately, no tissue was available from day 6 in experiment 2 for this assay. Viral load in terms of CD_50_ was calculated for each sample using the Reed-Muench method. Statistical differences were identified using a One-Way ANOVA followed by Tukey’s test for multiple comparisons and are highlighted by * (p<0.05) and ** (p<0.005). Of note, one sample of eyelid was damaged during extraction and there was not enough tissue available for this assay therefore in panel C there are only 4 birds for M41K-4/91(S).

The main site of *in vivo* infection for IBV is the upper respiratory tract including the trachea. Eyelid tissue has also been demonstrated to be a site of infection [48]. In both *in vivo* experiments the quantities of infectious progeny at day 4 pi were determined by titration from both tracheal and eyelid derived tissue supernatants in *ex vivo* TOCs (Fig 3). In both eyelid and trachea, infectious M41K-Beau(S) could be detected demonstrating that the Beaudette S results in infection of both tissues *in vivo*. No statistical differences were detected between M41-K and M41K-Beau(S) (Fig 3A, 3B, 3D and 3E). Differences were identified however between M41K-4/91(S) and the genomic backbone M41-K in eyelid tissue and also in comparison to the S donor strain 4/91 in tracheal tissue (Fig 3C and 3F). This may suggest whilst the insertion of the S gene from heterologous IBV strains may not impact the tropism within the upper respiratory tract, it may impact viral replication in cases where there are greater differences in amino acid sequence between the S proteins.

### Recombinant IBVs expressing the Beaudette S offer protection against challenge with M41

**Fig 4:**
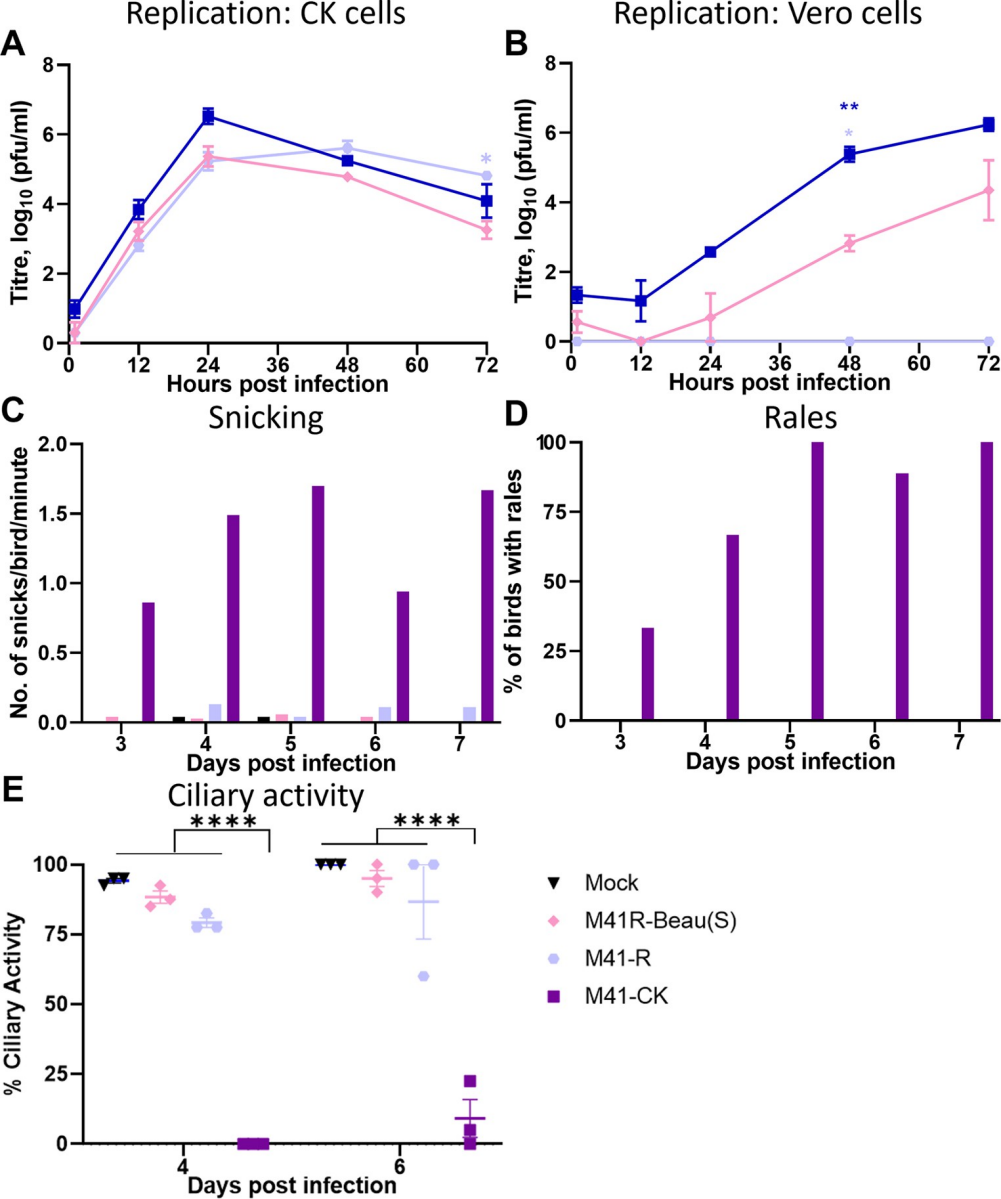
M41R-Beau(S) is attenuated *in vivo*. CK cells (A) and Vero cells (B) were inoculated with either 0.5 x 10^3^ PFU of M41R-Beau(S), Beau-R or M41-R (MOI ~ 0.001). Supernatant was harvested and titrated on CK cells. Error bars represent SEM of three independent repeats. Statistical differences were analysed using a Two-way ANOVA with a Tukey’s test for multiple comparisons. Differences identified are (A) Beau-R vs M41-R 72 hpi; (B) Beau-R vs M41-R, 24–96 hpi and M41R-Beau(S) vs M41-R and vs Beau-R at 48 hpi (*p<0.05, **p<0.005). (C- E) Groups of 12 SPF RIR chickens at 8 days of age were inoculated with 10^5^ PFU of M41R-Beau(S), M41-R or M41-CK or mock inoculated with PBS via the intraocular and intranasal route. Clinical signs were observed from day 3 to 7 post-infection. (C) The average number of snicks per bird per minute was calculated for each group based on counts from two persons. (D) The percentage of birds exhibiting rales in each group was calculated. (E) Trachea were extracted from three randomly selected birds on each day and sectioned into 10 x 1 mm rings. Each ring was analysed under a light microscope and assigned a score between 1 and 4 depending on the proportion of cilia beating. 1 = 25% beating, 2 = 50%, 3 = 75%, 4 = 100%. Each data point represents the average percentage ciliary activity of each bird. Error bars represent SD. Statistical differences were assessed using a Two-Way ANOVA followed by Tukey’s test for multiple comparisons and are highlighted by **** (p<0.0001).

To assess the immunogenic potential of the Beau-R S glycoprotein a homologous vaccine-challenge study was carried out. As the replication of Beau-R is temperature sensitive, a second rIBV was generated using the attenuated M41-R genomic background, which possesses the amino acid changes Pro85Leu in nsp 10, Val393Leu in nsp 14, Leu183Ile in nsp 15 and Val209Ile in nsp 16 [64]. The generation of a rIBV with a temperature sensitive backbone and an attenuating S protein allowed the investigation as to whether attenuation by multiple independent mechanisms impacted vaccine efficacy. *In vitro* analysis demonstrated that rIBV M41R-Beau(S) replicated in both CK and Vero cells and was attenuated *in vivo*, with no observed clinical signs and tracheal ciliary activity retained at both day 4 and 6 pi (Fig 4).

**Fig 5:**
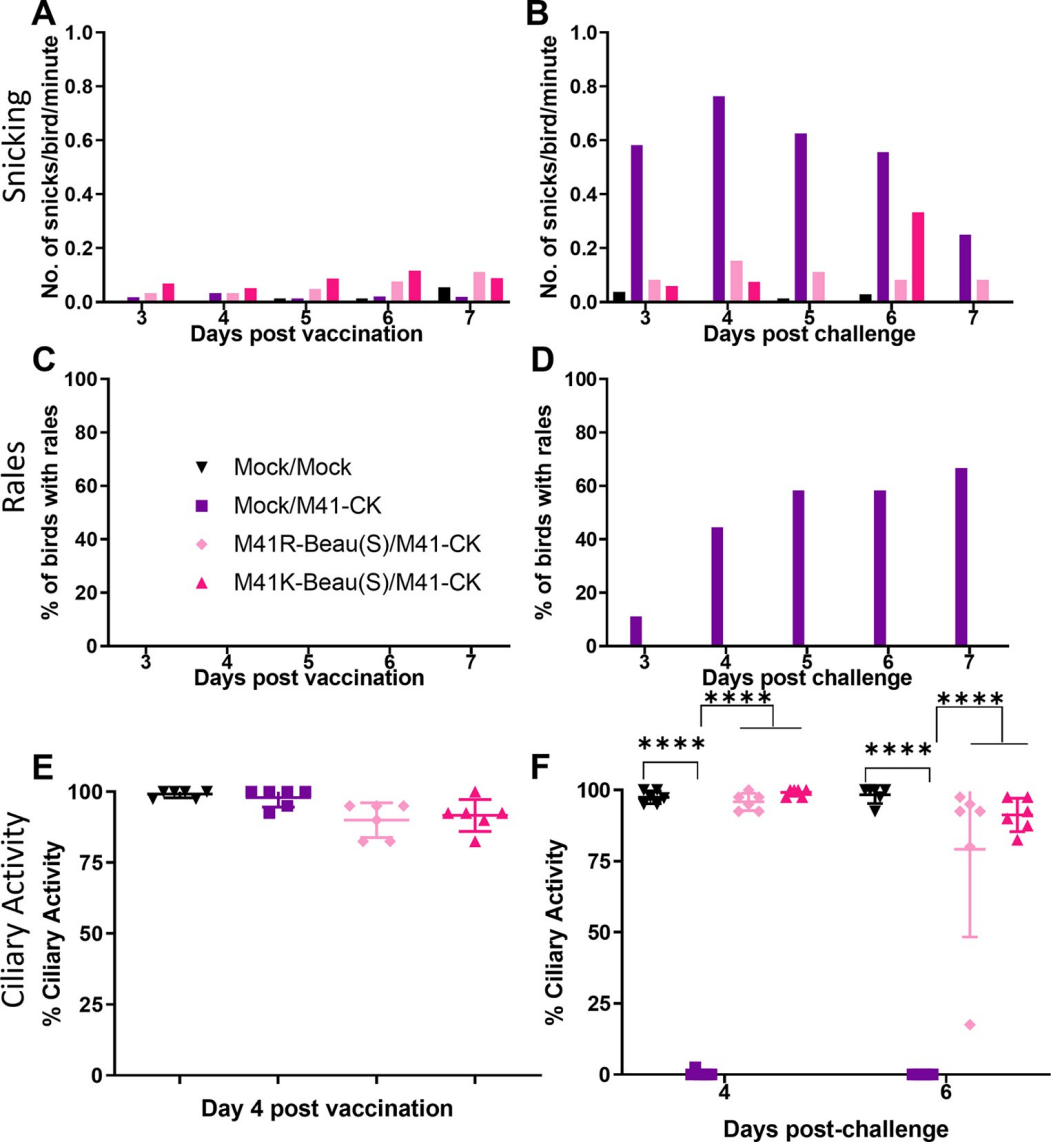
Vaccination with M41K-Beau(S) or M41R-Beau(S) protects against M41-CK challenge. Groups of 30 SPF RIR chicks were vaccinated at 8 days old with 10^4^ PFU of either M41K-Beau(S) or M41R-Beau(S) or mock vaccinated with PBS via the intraocular and intranasal route. At 27 days post-vaccination chickens were either challenged with 10^4^ M41-CK or mock challenged with PBS also via the intranasal and intraocular route. Clinical signs including snicking (A and B) and rales (C and D) were observed from day 3 to day 7 post-vaccination and day 3 to 7 post-challenge. Trachea were extracted from six randomly selected birds on day 4 post-vaccination (E), day 4 and 6 post-challenge (F) and sectioned into 10 x 1 mm rings. Each ring was analysed under a light microscope and assigned a score between 1 and 4 depending on the proportion of cilia beating. 1 = 25% beating, 2 = 50%, 3 = 75%, 4 = 100%. Each data point represents the average percentage ciliary activity of each bird. Error bars represent SD. Statistical differences were assessed using a Two-Way ANOVA followed by Tukey’s test for multiple comparisons and are highlighted by **** (p<0.0001).

Groups of 18 8-day-old RIR chicks were vaccinated via the intraocular and intranasal routes with 100 μl of PBS containing either 10^4^ PFU M41K-Beau(S) or M41R-Beau(S), or mock vaccinated. All groups were subsequently challenged 27 days later with 100 μl of PBS containing 10^4^ PFU M41-CK or mock challenged. Clinical signs were assessed from days 3 to 7 pv and pc (Fig 5). Very few snicks were observed following vaccination with either M41K-Beau(S) or M41R-Beau(S), with the numbers in each group comparable to the mock vaccinated group (Fig 5A). No rales were observed post-vaccination in any group. Ciliary activities were measured in the tracheas harvested from six randomly selected birds on day 4 post-vaccination and were found to be comparable between both vaccinated groups and the mock vaccinated group (Fig 5E). Following challenge with M41-CK, the M41K-Beau(S) and M41R-Beau(S) vaccinated groups, M41K-Beau(S)/M41-CK and M41R-Beau(S)/M41-CK respectively, displayed snicking comparable to both the mock vaccinated/mock challenged (Mock/Mock) group (Fig 5B). The only exception was day 6 pc in which snicking was slightly increased in the M41K-Beau(S)/M41-CK challenged group, however, the observed snicking was lower than the mock vaccinated/M41-CK challenge group. Rales were only observed in the mock vaccinated/M41-CK challenged group; no rales were observed in either of the rIBV vaccinated/M41-CK challenged groups (Fig 5D). On day 4 pc in all groups except the mock vaccinated/M41-CK challenge group, almost 100% ciliary activity was retained in all the birds examined (Fig 5F). On day 6 pc a slight reduction in the ciliary activities was observed in the M41K-Beau(S) vaccinated group, however, it still exceeded 75%. Interestingly, one of the M41R-Beau(S) vaccinated birds exhibited a reduced tracheal ciliary activity at 17.5%; the rest of the birds within the group exceeded 75% activity. This retention of tracheal ciliary activity alongside the lack of IBV-associated clinical signs in both the rIBV vaccinated groups demonstrates that both M41K-Beau(S) and M41R-Beau(S) can induce a protective response against M41 CK challenge. This observation demonstrates that the Beaudette S glycoprotein is both antigenic and immunogenic with the capability of producing a protective immune response, either from an attenuated or pathogenic M41 genetic background.

### Vaccination with rIBVs based on an M41 genetic background expressing the Beaudette S glycoprotein induced a robust antibody response

**Fig 6:**
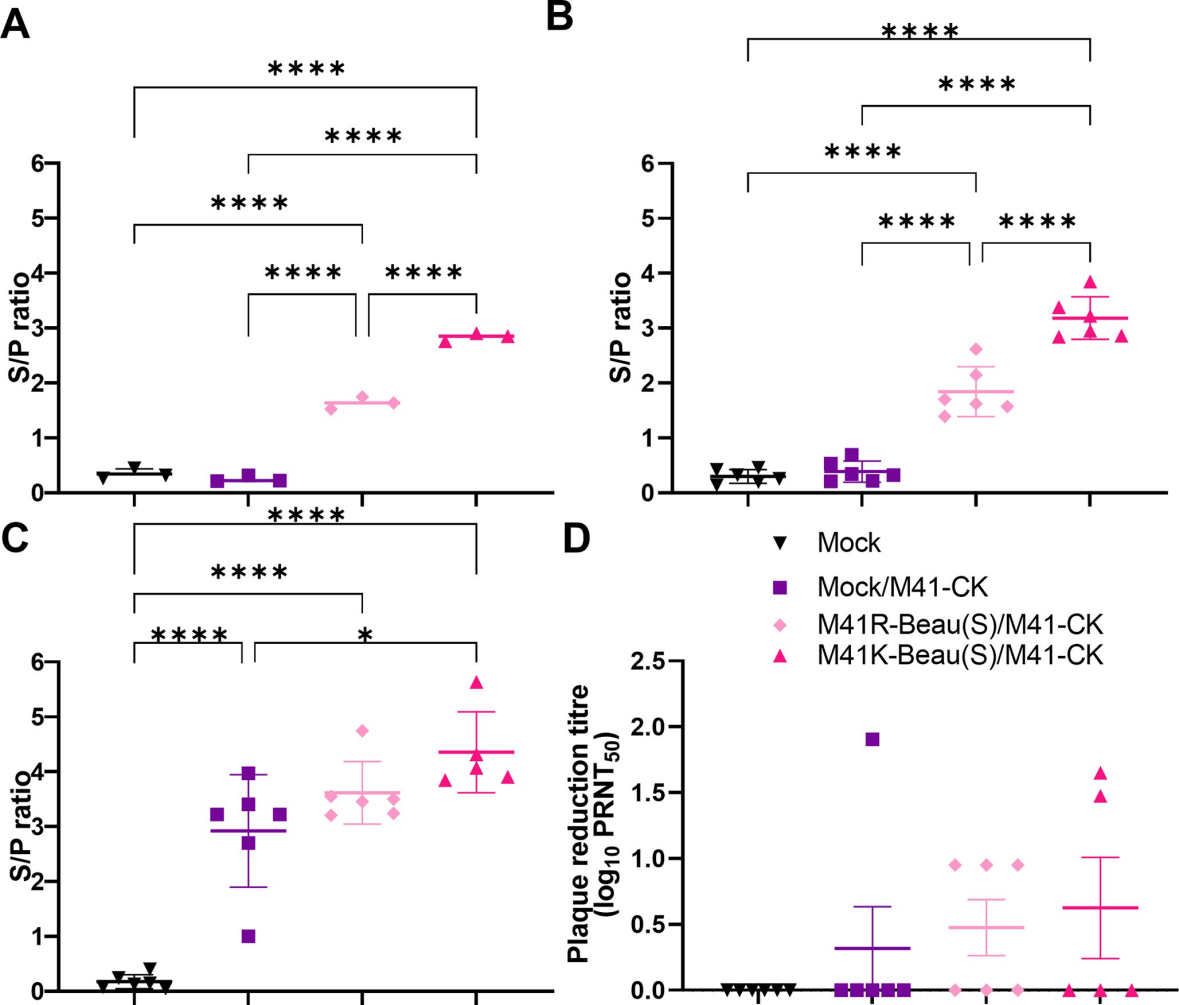
Vaccination with M41K-Beau(S) and M41R-Beau(S) induces a robust antibody response. Serum was harvested from chickens on day 14 post-vaccination (A) and days 4 (B) and 14 post-challenge (C) and diluted 1/80 for ELISA using the commercial BioChek IBV ELISA kit. Samples from each bird in a group harvested on day 14 post-vaccinated were pooled together due to the limited quantities of serum able to be collected. Samples were run in triplicate, and the average S/P ratio was calculated for each bird. S/P ratios for each independent repeat (A) or for each bird (B and C) are displayed with error bars representing the SD. (D) To assess the levels of neutralizing antibody, serum from day 14 post-challenge was serially diluted and incubated with 10^3^ PFU of M41-CK, followed by a plaque assay on CK cells to determine the plaque reduction neutralization titer (PRNT_50_). The PRNT_50_ values were calculated for each group using the Reed-Muench method. Average PRNT_50_ values for each bird are displayed, with error bars representing the SD. Statistical differences were assessed by one-way ANOVA, followed by Tukey’s test for multiple comparisons and are highlighted by * (p<0.05), *** (p< 0.0005) and **** (p<0.0001).

The humoral immune response to vaccination and challenge was assessed by both ELISA (Biochek) and virus neutralisation assays (Fig 6). On days 14 pv (Fig 6A) and day 4 pc (Fig 6B), significantly more IBV-specific antibodies were detected in both vaccinated groups compared to the mock vaccinated/M41-CK challenged group, indicating that vaccination with M41R-Beau(S) or M41K-Beau(S) had induced a humoral immune response. A significantly higher level of IBV-specific antibody was detected in birds vaccinated with M41K-Beau(S) than those vaccinated with M41R-Beau(S) suggesting differences in the ability of the two differing rIBV genetic backgrounds to elicit a humoral response. In support of this observation only the M41K-Beau(S) vaccinated/M41-CK challenge group, had statistically higher levels of IBV antibodies, at day 14 pc, in comparison to the mock vaccinated/M41-CK challenged group (Fig 6C). The presence of serum neutralising antibodies against M41-CK was assessed by determining the plaque reduction neutralization titre (PRNT_50_) for each bird at day 14 pc (Fig 6D). As expected, no neutralizing antibody against M41-CK was detected in the mock group. Neutralizing antibodies were only detected in one bird in the mock/M41-CK-challenged group. Virus neutralising antibodies were detected in both rIBV vaccinated groups, with neutralising antibodies detected in 3 of 6 birds in the M41R-Beau(S) vaccinated group and 2 of 5 in the M41K-Beau(S) vaccinated group. The presence of IBV specific antibodies in the rIBV vaccinated group, and specifically the presence of neutralising antibodies, suggests that the Beau-R S glycoprotein is capable of eliciting a robust humoral response.

## Discussion

Insertion of heterologous S genes into IBV has previously been shown to alter cell tropism and provide a degree of protection following both homologous and heterologous challenge [32, 46, 67, 70], demonstrating that S gene replacements or modifications may offer an avenue for the generation of rationally designed IBV vaccines. The S gene of the IBV strain Beaudette has favourable characteristics in terms of vaccine development as previous research using reverse genetics indicated that it was the S gene, and specifically the S2 subunit that confers Beaudette’s unique ability to replicate in Vero cells [46, 71]. Vero cells are licenced for vaccine manufacturing and are used for the production of vaccines against Ebola virus, Influenza virus and Smallpox [52]. Previous research modified a Beaudette based molecular clone, rIBV Beau-R [61] to remove the ability to replicate in Vero cells [46, 71], and as such contributory factors located elsewhere in the genome could not be conclusively ruled in or out. Our study uses a M41 based clone [56] with the aim of conferring the ability to replicate in Vero cells thereby conclusively confirming the ability of the Beaudette S gene to enable the ability to replicate in Vero cells to the previously non-permissive M41 strain (Fig 1D). This is a significant finding as the inability of the majority of IBV strains to replicate in cell culture has historically presented a barrier to the development of vaccines independent of embryonated hens’ eggs.

Prior to this study, the benefit of a rIBV expressing the Beaudette S protein was not clear as the Beaudette strain of IBV is considered poorly immunogenic [32, 39, 40]. Additionally, it was unknown whether a pathogenic rIBV expressing the S protein from an attenuated IBV strain would result in an attenuated *in vivo* phenotype. Research using the QX-like YN strain indicates a role for the S gene in attenuation [72]. The S gene of the pathogenic molecular clone, YN was replaced with the corresponding sequence derived from an attenuated YN strain in which attenuation was achieved via the traditional method of serial passaging in CEE [72]. The resulting clone was deemed to be apathogenic *in vivo* as characterised by a lack of clinical signs. Tracheal damage was still observed however and was comparable to the pathogenic clone from days 3 to 9 pi. Whilst encouraging, this research did not represent a heterologous S gene swap nor complete attenuation. In this study we have shown that replacing the S gene in the pathogenic rIBV M41-K with the equivalent sequence from the attenuated IBV Beaudette, generated a rIBV, M41K-Beau(S), that exhibits an attenuated phenotype *in vivo*, both in terms of clinical signs and tracheal ciliary activity (Fig 2). However, there was the possibility, that the observed attenuation resulted simply from the presence of a large heterologous S gene nucleotide sequence, rather than the Beaudette S sequence *per se*. We subsequently demonstrated that the replacement of the M41 S gene with an equivalent but more diverse nucleotide sequence from a pathogenic IBV, 4/91, resulted in a rIBV, M41K-4/91(S), which exhibited a pathogenic phenotype *in vivo* (Fig 2). The 4/91 S sequence shares only 81.7% amino acid identity with that of M41, compared to 95.4% between M41 and Beaudette, indicating that it is unlikely that the heterologous nature of the Beaudette S nucleotide sequence in an M41 genomic background was responsible for the attenuated *in vivo* phenotype observed by rIBV M41K-Beau(S).

Historical work had always indicated that Beaudette could not be used as a vaccine as it was too attenuated [12, 32, 39]. Although both the Beaudette and M41 strains of IBV belong to the same serotype, Massachusetts, there are amino acid differences between the two S glycoproteins. Previous research regarding cross protection between IBV strains has indicated unpredictability in vaccine efficacy, as few amino acid differences can result in limited cross protection [14, 26, 30, 31, 73]. Cross protection is a concern not only for IBV but also increasingly for SARS-CoV 2 as a consequence of continual viral evolution and variant emergence [36, 74]. With regards to this study, previous work had hypothesised that sequence differences in the S protein had negatively impacted Beaudette’s ability to induce a protective immune response against an M41 challenge [32, 39]. In this study vaccination of chickens with M41K-Beau(S) or M41R-Beau(S) resulted in protection against challenge with pathogenic M41 as defined by tracheal ciliary activity and lack of clinical signs (Fig 5). IBV-specific antibodies and virus-neutralising antibodies were detected in the serum from both M41K-Beau(S) and M41R-Beau(S) vaccinated birds post-challenge (Fig 6), indicating that Beaudette S glycoprotein is capable of eliciting a humoral response. It must be noted that several of the vaccinated/challenged birds did not score positive for the presence of neutralising antibody, a phenomenon that has been observed previously [64]. This may be the consequence of the sensitivity of the method of the virus neutralisation. The presence of humoral immunity, including that of neutralising antibodies, however, is generally considered unreliable as a correlate of protection [9, 75]. Significantly higher levels of IBV-specific antibody were detected in birds vaccinated with M41K-Beau(S) over M41R-Beau(S) and it must be noted whilst post-challenge all birds vaccinated with M41K-Beau(S) met the standards set by the European Pharmacopoeia, only 4 of 6 in the M41R-Beau(S) group did. Differences in protection offered between M41K-Beau(S) and M41R-Beau(S) may correlate to differences in replication capacity as the incorporation of Pro85Leu in nsp 10 and Val383Leu in nsp 14 has been demonstrated to impart a temperature sensitive replication phenotype, although this previously did not impact vaccine efficacy [64]. Reduced vaccine efficacy as a possible consequence of over attention has been demonstrated previously [11].

Existing live-attenuated IBV vaccines are known to cause some clinical signs and even damage to tracheal ciliary activity and therefore vaccination with M41K-Beau(S) or M41R-Beau(S) may offer advantages over the currently used Massachusetts vaccines, H52 and H120 [10, 76]. Furthermore, traditional methods of serial passage to obtain live-attenuated vaccine viruses are associated with a risk of reversion to virulence, with studies identifying that only a few consensus level nucleotide sequence changes are needed to occur [60]. Attenuation achieved through the insertion of the entire S ectodomain sequence accompanied by the clonal origin of the rIBV which eliminates the risk of “back selection” means that reversion to virulence through spontaneous mutation is unlikely. Whilst our previous work has investigated methods of targeting the rIBV replicase gene for rational attenuation [56, 65] this study identifies the S gene as an alternative target, and one that can provide advantageous characteristics such as the ability to replicate in cells licenced for vaccine manufacturing. The Beaudette S gene may therefore offer a significant promising avenue for the development of recombinant IBV vaccine viruses.
